# Navigating the light and shadow of scientific publishing faced with machine learning and generative AI

**DOI:** 10.1002/ejp.4736

**Published:** 2024-10-03

**Authors:** Federico Palmisani, Daniel Segelcke, Jan Vollert

**Affiliations:** ^1^ Department of Clinical and Biomedical Sciences, Faculty of Health and Life Sciences University of Exeter Exeter UK; ^2^ Department of Anaesthesiology, Intensive Care and Pain Medicine University Hospital Muenster Germany

## Abstract

**Background:**

The public release of ChatGPT in November 2022 sparked a boom and public interest in generative artificial intelligence (AI) that has led to journals and journal families hastily releasing generative AI policies, ranging from asking authors for acknowledgement or declaration to the outright banning of use.

**Results:**

Here, we briefly discuss the basics of machine learning, generative AI, and how it will affect scientific publishing. We focus especially on potential risks and benefits to the scientific community as a whole and journals specifically.

**Conclusion:**

While the concerns of editors, for example about manufactured studies, are valid, some recently implemented or suggested policies will not be sustainable in the long run. The quality of generated text and code is quickly becoming so high that it will not only be impossible to detect the use of generative AI but would also mean taking a powerful tool away from researchers that can make their life easier every day.

**Significance:**

We discuss the history and current state of AI and highlight its relevance for medical publishing and pain research. We provide guidance on how to act now to increase good scientific practice in the world of ChatGPT and call for a task force focusing on improving publishing pain research with use of generative AI.

## 1 DO NOT LET THE PERFECT BE THE ENEMY OF THE GOOD

When we learn maths at school, we get used to the omnipresence of absolute and unequivocal answers to problems: two plus three equals exactly five. It does not only not equal four or six but also not 4.9999 or 5.0001. It also always equals five, independently of by whom, when and where it is calculated. This type of math is called analytical, and as it is what we use most in our daily lives, it is what we consider to be standard.

Computers operate on a different type of maths due to their principal structure. This structure does not allow for them to solve equations analytically (meaning, solving for x), they can only perform iterative models. Iterative or computational maths differs so that the solution to two plus three in iterative terms is unlikely to ever be five. Instead, it will approach five, depending on how much computing power is allocated. In most immediate and simple terms, it may report the sum of two and three to be something between four and six. Using more computing power, we can now generate a sequence of increasingly accurate solutions (*n* th approximation), where each subsequent approximation is based on, and more precise than, the previous one. Using a predefined precision, we can stop when we are satisfied that our estimate is close enough for our purposes. In our example, we might be content with the approximation that the sum of two and three equals something between 4.9999 and 5.0001 (for illustrative purposes, this is a simplified example: in reality, computers can do basic addition, but are unable to perform differentiation and integration. The principal remains the same (Gill, 1991)). Iterative methods are particularly useful when direct solutions are too complex.

An analogy of solving problems iteratively is how we move through traffic: when we want to get from home to work, we will start with the shortest route. Once we encounter heavy traffic though, we might adopt our route to a longer, but potentially faster one, based on real‐time data we are gathering. Similarly, we can understand the solution strategy of computing to begin with an initial solution, the collection of feedback from real‐time data, and reassessment and adjustment of the initial position, repeating these steps until the solution is within acceptable uncertainty boundaries. Navigation apps have mastered the problem of finding near‐perfect routes, a problem for which there is no known perfect solution (Biggs et al., 2006).

Iterative mathematics for solving complex equations has been around for a long time. It was first described by the Persian mathematician Jamshid al‐Kashiin in his work ‘The Key to Arithmetic’ in the year 1429 (Bagheri, 1997). However, before the invention of the computer, there were few applications, considering it is labour expensive (and very dull) to perform the same calculation hundreds of times to get a slightly closer estimate. However, repeating the same task hundreds, thousands or millions of times over is something computers excel at (Henrici, 1993). With computing power having increased exponentially for decades, for many problems, iterative solutions are now so precise that they are virtually indistinguishable from perfect solutions.

For more complex and computationally challenging problems, we are still working on improving performances. In some of the most demanding instances, machine learning has shown to be exceptionally helpful in finding approximate solutions (Jumper et al., 2021).

## 2 HOW CAN WE TEACH WHAT WE DO NOT UNDERSTAND?

While we have long used computers to iteratively solve problems, we can only use traditional computing if we are able to phrase the problem into a set of repeatable steps that the computer can perform—this process is known as an algorithm. In reality, however, there are problems that we cannot easily phrase into a set of clear rules, or the maths resulting are practically unsolvable (Badrulhisham et al., 2024; Biggs et al., 2006). Think, for example, you were asked by an alien (or a robot) what the difference is between a cat and a dog. For any rule you will be able to think up, there will be exceptions: dogs are usually smaller, until you think of a lion and a dachshund. Dogs are usually fluffier, until you think of a lion and a dachshund. Dogs are usually physically stronger, until you think of a lion and a dachshund. At this point, the robot or alien might ask if those two should switch kingdoms.

Machine learning algorithms are a set of methods that can produce extraordinary results on a set of questions for which we do not necessarily need perfect solutions, but can accept increasingly better approximations, and we cannot phrase a fixed set of rules, as we ourselves do not fully understand what the rules are (or when we do, they are so complex that they cannot be calculated, like protein folding or planetary movements: we know the forces at hand and have equations, but they cannot be solved in this universe (Dill et al., 2008)). Unlike traditional algorithms, machine learning algorithms do not rely on predefined rules but rather learn through examples. This is similar to how humans learn intuitively, using trial and error: the reason why we cannot define a set of rules separating cats from dogs is that there is none, and we have only learned to separate them by looking at thousands of pictures or real‐life examples (Tulis et al., 2016). Therefore, using appropriate learning data sets is one of the most crucial aspects of successful machine (as well as human) learning (Badrulhisham et al., 2024).

## 3 A SURPRISINGLY OLD FIELD

OpenAI released the inaugural edition of ChatGPT, an abbreviation for ‘Generative Pre‐trained Transformer’, on 30 November 2022 (OpenAI, 2024), thereby initiating the existing hype and wave of public interest in artificial intelligence (AI). However, the field of AI‐research goes back many decades, and even neural networks as a method for achieving AIs is more than 40 years old (Hopfield, 1982), while the mathematical models behind it, other methods and the wider concepts have existed for more than eight decades (Cover & Hart, 1967; McCulloch & Pitts, 1943).

It is important to note that throughout the history of AI, periods of significant advancements were often followed by what has been termed an ‘AI winter’. These were prolonged phases where AI development failed to meet expectations or overcome inherent challenges, resulting in minimal progress for over a decade on two separate occasions (Russell et al., 2003). When we extrapolate from the recent, undeniably substantial leaps forward enabled by generative AI, we should remember that previous overoptimism has proven misguided.

## 4 DEEP LEARNING AND GENERATIVE AI

While there are no generally agreed definitions, currently, the term AI is mostly used to describe the automation of complex tasks we would generally attribute human intelligence to (Luger, 2004). Machine Learning is the set of methods we use to achieve an AI (Badrulhisham et al., 2024). In recent years, the most prominent and capable among these has become deep learning (LeCun et al., 2015), which is based on neural networks (McCulloch & Pitts, 1943).

Neural networks mimic biological processes, as they are based on the structure of the simplest possible neuronal system (an input layer, a hidden processing layer and an output layer). With multiple interconnected nodes, the capability of the network grows with its complexity and number of hidden layers. A node simply will either allow a signal to move forward or not (inspired by a post‐synaptic potential either reaching the activation threshold at the axon hillock or not (Badrulhisham et al., 2024)). In deep learning, neural networks are constructed consisting of hundreds, thousands or millions of nodes in multiple hidden layers (LeCun et al., 2015).

Deep learning approaches serve as the basis of chatbots like ChatGPT, which rely on large language models (LLMs) (OpenAI, 2024). LLMs use vast amounts of text to learn, and are generative (i.e. they can generate new text from their input, unlike, for example, Google, which can only show you existing texts). These models are at the heart not only of ChatGPT but also Google's Gemini (previously known as Bard) or Microsoft's Copilot, all three of which serve as virtual assistant interfaces to the complex machinery that has learned from the human knowledge and interactions published on the internet.

It is this generative component that will likely have the most disruptive impact (Lock, 2022). This is not just because text (or image, or video or music, etc.) generation up to this point was not only an almost exclusively human skill, which we ascribe intelligence to and which we use to assess and grade intelligence and competence on. It also touches the core of our collective identity, as creating and creativity are something so intrinsically human that we philosophically consider it to be a pillar of what it means to be human (and sentient) in the first place (Kelly, 2022; Nussbaum, 2018).

## 5 THE DYSTOPIAN VIEW: RISKS OF GENERATIVE AI IN PUBLISHING AND READING SCIENTIFIC LITERATURE

### 5.1 A rapidly moving field

With the public release of ChatGPT just over 1.5 years ago, it will still take significant time for the scientific community to catch up in performing studies and to construct guidelines and standards under which AI use will be practical and scientifically justifiable. So far, only few guidelines are ready to use (Chan, 2023; NCEE, 2024), while others are in development and due to be released this year (UNESCO, 2024) with overall guidance published (Miao, 2023). The uncertainty towards this new field and its pace of development can be exemplified by a study (Chan, 2023), where a survey was performed among 457 students and 180 teachers from different disciplines across Hong Kong universities investigating, among other aspects, the use of generative AI. Both students and teachers reported low experience with generative AI, which appears to be a surprising read in an article explicitly covering ChatGPT. However, the article was submitted in March 2023, implying that data collection will have mostly taken place before the public widespread adoption of generative virtual assistants. When analysing the literature, this knowledge gap needs to be taken into account considering the pace of recent developments in generative AI.

Rapid, massive changes in the field, however, will make it difficult for regulation to keep pace with developments (Miao, 2023). This is a generally acknowledged problem with AI that affects teaching specifically. Changing policies and establishing systems to enforce them can take years, a time by which the adopted policies might be obsolete and outdated. The dilemma of policy catching up with technology is in itself not new and can be compared, for example, to illegal doping in professional sports.

### 5.2 Perpetuating bias

As stated above, machine learning learns by example. LLM‐based chatbots have been trained using the collective knowledge of the internet, a uniquely rich source of a kind that has not been present at any other time in human history. However, the internet is not always a safe space, nor is it free from falsehoods or disinformation, and it is heavily biased in favour of the Global North.

Therefore, using appropriate learning data sets is one of the most crucial aspects of successful machine learning, and biased input datasets have led to the failure of many AIs. For example, the widely used photo database ImageNet. China and India, however, despite comprising more than a third of the world's population, constituted just 3% of depicted people on ImageNet (Shankar et al., 2017). Since AIs aim to optimize their accuracy, they frequently ignore minorities (Zou & Schiebinger, 2018) and will perpetuate such information to the user.

While we tend to believe in the neutrality of computational results, by learning from human annotated data, AIs have learned and adopted our own biases. For instance, internet search algorithms have been shown to propagate gender stereotypes (Vlasceanu & Amodio, 2022), and AI‐based decision making in US hospitals disadvantaged Black patients, preventing them from accessing necessary care and treatment a similar White patient would have received under similar circumstance (Obermeyer et al., 2019). As a scientific community, we must be aware not to be too trusting in the objectivity of AI and enable policies and guidelines to reduce the aforementioned biases.

This has particular relevance to pain research and treatment, as pain as an inherently subjective experience is often diminished in the absence of visual or otherwise obvious signs of injury (De Ruddere et al., 2013). While the International Association for the Study of Pain (IASP) rejects the use of biomarkers to prove or disprove someone's pain (Davis et al., 2017), it is still commonly discussed that in the near future, ‘pain lie detectors’ can be used to objectify pain measures (Lalkhen, 2023). This falls under the category of the above‐described belief in the objective machine, that will, however, learn from flawed data. The experimental setting used to train the machine will not be representative of the real‐life testing situation (Morais et al., 2022), and participants in experimental studies tend to overrepresent white, highly educated, affluent parts of society (Letzen et al., 2022). In reality, though, pain is often more prevalent in minorities of societies, including those racialized (Mathur et al., 2022), leading to systematic underestimation of patients' pain through healthcare providers (Seers et al., 2018) and under provision of much needed pain care (Craig et al., 2020).

### 5.3 Hallucination and paper mills

One significant challenge associated with LLM‐based virtual assistants is their tendency to deliver responses that sound correct, even if there is no valid answer or the chatbot lacks the information needed to provide an answer. This problem is frequently called hallucination (Maynez et al., 2020), as it creates false events and makes them appear as real. Typically, chatbots do not disclose the origin of their responses (and in reality, it is highly complex and a significant area of research to develop AI systems that can provide transparent and comprehensible decision making). As a consequence, there is significant concern that LLM‐based chatbots will contribute to the proliferation of false information on the internet (Emsley, 2023). This could transform a repository of human knowledge into an impenetrable place, rendering it impossible to retrieve valuable information (Miao, 2023). In scientific research, it could turbocharge the existing issue of scientific databases being flooded by false scientific publications propagated by ‘paper mills’ (Emsley, 2023).

An important role for scientific journal editors in the context of generative AI will be to avoid this dystopia. While it will be mostly on international policy makers and AI developers to reduce the amount of generated false information, it will be our responsibility as editors to work as hard as we can to enable systems to recognize the difference between true and true‐sounding information. Governing bodies like UK's AdvanceHE work on providing workshops to teach identifying hallmarks of true versus true‐sounding information (AdvanceHE, 2024).

When using generative AI, we must be aware of several factors, including their lack of neutrality, what the source of their material is, and how—or if—we can overcome their biases and contribute to the decolonisation of medicine by avoiding an overreliance on discriminatory outputs and further perpetuating historic stereotypes.

## 6 THE UTOPIAN VIEW: CHANCES OF GENERATIVE AI IN PUBLISHING AND READING SCIENTIFIC LITERATURE

### 6.1 Face the inevitable

Despite the risks outlined above, it is generally recommended that ethical and responsible use of LLM‐based chatbots and generative AI should be encouraged (Miao, 2023; NCEE, 2024). This simply acknowledges that an AI‐free workspace is unattainable or unenforceable (Chan, 2023). While there are tools and procedures to detect AI‐generated texts, we need to keep in mind that what we are currently facing is the infancy of generative AI: their ability to mimic human texts will quickly evolve and is already outpacing our ability to detect it (Heikkilä, 2022). We will, therefore, have to learn to live with generative AI and adopt our practices accordingly.

### 6.2 Increasing reach and improving readability

Writing scientific manuscripts is a complex skill not much taught in our higher and scientific education. Conveying complex information in a condensed format to various levels of readerships requires careful tuning of terms, balancing simplicity and accuracy. The complexity becomes apparent when you consider the target audiences of a scientific article. These include professionals specializing in the research field, professionals in related fields, the wider scientific community, regulatory authorities, lay people with a particular interest and general lay people. People educated to tune their writing for each of these audiences are not typically active researchers (and be it simply as each is a career of its own). Breakdowns in communication may have contributed to an often held ‘ivory tower’ view of the scientific community in the general public (Adler et al., 2018), a gulf that we want to cross.

Leveraging generative AI to enhance text readability and adapt it to the targeted audience represents a significant opportunity afforded by this technology. A once‐written scientific manuscript will not need time‐consuming editing for readability anymore, and with a well‐trained and well‐prompted AI, multiple versions of the same article can be generated almost instantly for the multiple levels of possible readership described above. This process, which can be interpreted as a new form of retranslation (Monti, 2024), could contribute to a more effective science communication, enabling different types of readers to grasp complex concepts at different levels. BioRxiv is currently trialling an AI‐generated summary of the published preprints, where the reader can use a slider from ‘general’ to ‘expert’ level to increase the reach of preprints (BioRxivs, 2024). This is a new and learning system, and authors still have the option to adjust the summaries if they find the automated version to be misrepresenting, showing the importance of human counter checking.

This can be particularly relevant for pain as a highly multidisciplinary field. Psychologists, physicians (anaesthetists, neurologists, surgeons, general practitioners), physiotherapists, sociologists, basic and clinical neuroscientists all speak their own languages (Dong & Bäckryd, 2023) but need to work together to fill the bio‐psycho‐social model of pain with life (Raja et al., 2020). Pain also has a large and engaged community of those with lived experience, adding an additional layer of much needed communication (Haroutounian et al., 2024).

### 6.3 A babel fish on your phone: Instant translation of full texts

English is the lingua franca of scientific writing, while most researchers (including the authors) are not native English speakers. As non‐natives, our language skills may approach but never reach the quality of native speakers. The nuances of one term over the other, how it is understood in one part of the world versus another, and cultural aspects behind semantics are often lost.

Moreover, there can be the issue of sheer vocabulary: using a self‐assessment online survey, a study conducted by the Economist found that the average native test‐taker had an English vocabulary of 20,000–35,000 words (Greene, 2013). Compare this to the common vocabulary size of foreign test‐takers, which is just 4500 words (less than the average native 4‐year‐old participant, which was around 5000 words). While foreign participants living abroad more than doubled their vocabulary, it was still just similar to that of a native 8‐year‐old (around 10,000 words). As these are self‐selected participants, the amount of vocabulary effectively known and used by non‐native speakers drawn from this is probably overestimated. A study from Taiwan found that of 166 participants studying English for 5 years, less than half knew the 1000 most common words in English and only 16% knew the 2000 most common words (Webb & Chang, 2012). This reflects their lexical knowledge at the end of their 5‐year study period, and if not practised, most of this knowledge will be lost in a short time after studying ends.

In the academic community, this is acknowledged to be a source of disadvantage for non‐native English speakers (Pérez Ortega, 2020). At best, this means that a second language speaker will take slightly longer, look up the occasional word, and be a little more tired after reading a scientific article; while at worst, it will mean that they cannot communicate their ground‐breaking work to the community. A meta‐analysis showed that articles written by non‐native English speakers are 2.5 times more likely to be rejected than those written by native speakers (Amano et al., 2023).

Generative AI has the ability to address these issues by demonstrating exceptional proficiency in instantly translating or writing grammatically correct texts to its native language. The process is not perfect, and various AIs have been more or less successful, while cultural nuances are still often lost. Still, the application DeepL has focused specifically on this aspect and can be used along with other AI chatbots to enable communication and dissemination on a much more level playing field between native and non‐native English‐speaking researchers.

AIs use of consistent terminology and language as compared to the diversity of individual translators can also be seen as an advantage (Boulanger, 2024). However, human supervision will be important due to the AIs tendency to create true‐sounding, but fabricated texts (Emsley, 2023). But even if AI‐translated texts are proofread by professional translators, it has been shown that this was already increasing translators' productivity by about 20% in 2006—long before the current leaps ahead (Boulanger, 2024).

Not only for communicating science but also for communicating pain language can form a barrier. Due to its invisible nature, pain must be effectively communicated, which is less possible in non‐native languages (Mustajoki et al., 2018). The fact that ethnic minorities are provided with less access to pain management has been linked to language barriers in multiple instances (Hollingshead et al., 2016; Nguyen et al., 2005; Rambachan et al., 2023). While we in no way want to state that racial and ethnic as well as other stereotypes that lead to biased belief of patients and treatment of pain will disappear if language as a barrier is removed, it might contribute to reducing the effect of these.

## 7 AN UNCLEAR WAY FORWARD

Generative AI will have a transformative effect on how we communicate, both with each other and with our resources. It has the capacity to disrupt a wide range of fields, from computer coding to filmmaking. Scientific publishing is one of the affected fields, and it will be partly our job to ensure that it changes science for the better, not worse. Just how to enable this positive change is a much trickier question, for which we will need policy makers, AI researchers, editors, publishers, reviewers and scientists to come together and develop comprehensive, practical and enforceable guidelines for usage of generative AI in scientific research and publishing.

## 8 WHAT TO DO NEXT

### 8.1 What you can do now

All of us should clearly acknowledge the use of AI‐powered generative tools in our manuscripts. This should be specific, including the specific tool and the specific use. For example, rather than using the generic statement ‘AI tools were used in the generation of this work’ we should declare, for example, ‘ChatGPT was used to assist the generation of R‐code for the analysis of the data. ProWritingAid was used to improve readability and language, including grammar’.

We need to stay conscious about the limitations of generative AI, in particular its complete absence of true substance knowledge. All code or text generated or corrected by AI needs to be carefully proofread by subject experts to avoid true‐sounding but factually false information seeping into our works (Hosseini et al., 2023).

Similarly, we must avoid unfounded trust in the neutrality of the machine. Initiatives like ‘confronting racism in pain research’ (Hood et al., 2022; Letzen et al., 2022; Morais et al., 2022) will continue to be needed to avoid training our AIs on biased data, and listening to patients can and must never be replaced by looking at AI predictions (Davis et al., 2017).

### 8.2 What editorial boards and scientific societies should do soon

Editorial boards will need clear and enforceable guidelines on the use of generative AI. This has happened speedily in the last months, and many journals have followed organizations like the association of medical editors (WAME, 2024) and the Committee on Publication Ethics (COPE, 2024) agreeing on the two baseline principles that (a) use of AI must be comprehensively acknowledged, and (b) AI does not under any circumstances fulfil authorship criteria, as all content generated must be checked by humans, who need to carry full responsibility of all code, data and text. This has been adopted by large publication families like Nature (Nature, 2023) and JAMA (Flanagin et al., 2023), as well as by this journal (European Journal of Pain, 2024).

Enforcing these policies, however, might prove much harder and will be a race of arms, in which, traditionally, the policing arm tends to lag behind (Heikkilä, 2022). Thus, we should not rely on technological tools purely to uncover fabricated results and texts or other misuses of generative AI though they can and should be developed and will be helpful in many, if the less sophisticated cases (WAME, 2024).

To increase trust in submitted original work, likely a stronger focus on the preregistration of studies, providing of full data sets, and similar means reducing the likelihood of fabrication of articles will become important. Trust in research is pivotal and is not just questioned on the basis of AI use, though this may turbocharge the number of fabricated articles (Emsley, 2023). The Enhancing Trustworthiness in Pain Evidence network has compiled recommendations tailored to pain research, covering the domains methodological rigour; openness and transparency; balanced communication; data authenticity; equity, diversity and inclusion; and patient and public involvement and engagement (O'Connell et al., 2024). As hopefully made clear above, generative AI will affect each of these domains in specific ways, so adhering to this framework to develop and assess original work will be crucial.

Topical review articles will be much easier to generate than original studies, posing an even greater thread to this article form—potentially reinforcing the trust‐based system of commissioned reviews rather than unsolicited submissions. In some ways, it might be questioned whether topical reviews will still be needed, if generative AI can provide hyper‐specific reviews on any topic almost instantaneously. The role of the topical review author might move from primary writer to fact‐checker over time. For this to be the case, however, the risk of hallucination and the ability to provide sources of generative AI will need to increase significantly, and likely specific generative AIs will need to be developed that focus on scientific rather than general applications, similar to how we use PubMed rather than Google to search for medical publications as it provides a somewhat trusted filter.

We will need to face the situation head on, one reason being to shift incentives. If we promote an ethical way of using generative AI, we will reduce the risk of abuse. To achieve this, we call on pain journal editors and societies to form a task force on the topic of the responsible use of generative AI in pain research. Only then can we make sure that all relevant groups, from those with lived experience to researchers, from practitioners to basic scientists, have a tailored framework to work with.
